# Ethnicity and survival after a dementia diagnosis: a retrospective cohort study using electronic health record data

**DOI:** 10.1186/s13195-022-01135-z

**Published:** 2023-03-29

**Authors:** Melissa Co, Christoph Mueller, Rosie Mayston, Jayati Das-Munshi, Matthew Prina

**Affiliations:** 1 Department of Health Service and Population Research, Institute of Psychiatry, Psychology & Neurosciences, King’s College London, David Goldberg Centre, De Crespigny Park, London, SE5 8AF UK; 2 South London and Maudsley NHS Foundation Trust, London, UK; 3 Department of Psychological Medicine, Institute of Psychiatry, Psychology & Neuroscience, King’s College London, London, UK; 4 Department of Global Health & Social Medicine, Faculty of Social Science & Public Policy, King’s College London, London, UK; 5 ESRC Centre for Society and Mental Health, King’s College London, London, UK; 6 Population Health Sciences Institute, Faculty of Medical Sciences, Newcastle University, Newcastle, UK

## Abstract

**Background:**

Individuals from minority ethnic groups in the UK are thought to be at higher risk of developing dementia while facing additional barriers to receiving timely care. However, few studies in the UK have examined if there are ethnic disparities in survival once individuals receive a dementia diagnosis.

**Methods:**

We conducted a retrospective cohort study using electronic health record data of individuals diagnosed with dementia from a large secondary mental healthcare provider in London. Patients from Black African, Black Caribbean, South Asian, White British, and White Irish ethnic backgrounds were followed up for a 10-year period between 01 January 2008 and 31 December 2017. Data were linked to death certificate data from the Office of National Statistics to determine survival from dementia diagnosis. Standardised mortality ratios were calculated to estimate excess deaths in each ethnicity group as compared to the gender- and age-standardised population of England and Wales. We used Cox regression models to compare survival after dementia diagnosis across each ethnicity group.

**Results:**

Mortality was elevated at least twofold across all ethnicity groups with dementia compared to the general population in England and Wales. Risk of death was lower in Black Caribbean, Black African, White Irish, and South Asian groups as compared to the White British population, even after adjusting for age, gender, neighbourhood-level deprivation, indicators of mental and physical comorbidities. Risk of death remained lower after additionally accounting for those who emigrated out of the cohort.

**Conclusions:**

While mortality in dementia is elevated across all ethnic groups as compared to the general population, reasons for longer survival in minority ethnic groups in the UK as compared to the White British group are unclear and merit further exploration. Implications of longer survival, including carer burden and costs, should be considered in policy and planning to ensure adequate support for families and carers of individuals with dementia.

**Supplementary Information:**

The online version contains supplementary material available at 10.1186/s13195-022-01135-z.

## Introduction

In the UK, older adults from Black Caribbean, Black African, and South Asian minority ethnic groups are thought to be at higher risk for developing dementia, and rates of vascular and early-onset dementias in particular are thought to be higher in Black Caribbean, Black African, and White Irish groups [1–4]. At the same time, structural and systemic racism, negative experiences with health services, and unclear referral pathways are barriers for minority ethnic individuals accessing dementia services [5–8]. Black and South Asian individuals with dementia have also been found to have higher levels of cognitive impairment upon diagnosis, implying that they may receive diagnoses later than comparison groups [9].

Given the disparities in risk and access to services experienced by minority ethnic older adults with dementia, it is unclear whether there are disparities in clinical outcomes. There have been few studies on survival after a dementia diagnosis which focus on mortality in specific ethnic groups or ethnic inequalities in survival, particularly in the UK. Previous studies assessing race/ethnicity and survival after dementia diagnoses in the USA and UK have suggested that minority racial/ethnic groups in the US may experience longer survival in dementia. These findings have been attributed variously to poor data quality (death is less well recorded/linked in minority ethnic groups), cohort selection effects (higher mortality rates due to health disparities at younger ages results in a healthier older population), and migration (immigration requires individuals to be relatively healthier, particularly if moving long distances, and in later life, older adult immigrants may move back to their country of birth, and their deaths may not be observed in studies) [10–12]. However, these hypotheses are not as well studied in the UK for dementia, and many studies do not focus on ethnicity specifically but rather include it as a confounder.

Here, we examine excess mortality and differences in survival across ethnic groups in a population of older adults with dementia from a South London secondary care mental health provider. We first calculate the excess mortality experienced by individual ethnic groups in our cohort as compared to the general older adult population in England and Wales. We then analyse whether factors such as emigration out of the cohort may account for any differences in survival between ethnic groups, as well as younger age at diagnosis, different dementia typology, and presence of other clinical comorbidities.

## Methods

### Participants and setting

We used data from South London and Maudsley Trust’s (SLaM) Clinical Record Interactive Search (CRIS). SLaM is a large secondary care mental health provider in southeast London, UK, which serves an ethnically diverse catchment area of 1.3 million residents [13]. Since 2007, anonymised electronic health record (EHR) data from SLAM have been made available for research via the CRIS application, which includes data from structured fields in the clinical record as well as unstructured text from clinical documents and other correspondence [13]. CRIS has received ethical approval from the Oxford Research Ethics Committee C, reference 18/SC/0372. CRIS can also be linked to data from death certificates in England and Wales, which is provided by the Office of National Statistics.

Our cohort consisted of patients 65 years of age or older in CRIS with an incident diagnosis of dementia over a 10-year period between 01 January 2008 and 31 December 2017. Patients were followed up until death or 31 December 2017.

Dementia was defined as having a diagnosis of F00* (dementia in Alzheimer’s disease), F01* (vascular dementia), F02* (dementia in other diseases classified elsewhere), F03* (unspecified dementia), and G30* (Alzheimer’s disease) in a structured diagnosis field. We also included mentions of one of the following terms in unstructured text: “F00”, “F01”, “F02”, “F03”, “G30”, “dementia”, “Alzheimer’s disease”, “Alzheimer’s”, “vascular dementia”, and “mixed dementia”. Diagnoses in unstructured fields were identified using a General Architecture for Text Engineering (GATE) natural language processing application [13]. Based on a test sample of 100 patients for whom we manually checked the unstructured text documents, we found that using these terms in the GATE algorithm yielded a 94% positive predictive value for identifying patients with dementia, and a 97.2% positive predictive value for identifying documents that indicate dementia diagnosis. We expected that most patients in our cohort would also have a diagnosis in a structured field as well.

As patients may receive multiple diagnoses in their records, we additionally determined the patient’s dementia subtype using an algorithm shown in Supplementary Fig. 1.

### Measures

Date and cause of death were ascertained by linkage to Office of National Statistics (ONS) Mortality data, which includes data from death certificates across the country and includes underlying cause of death using ICD-10 codes.

Ethnicity variables were derived from the patient record, which includes 16 ethnicity groups derived from ONS categories [14]. At the time of referral to SLaM services, patients are asked to self-identify with one of these ethnic groups. Due to very small sample sizes, we combined South Asian (Indian, Pakistani, Bangladeshi) ethnicity groups together as per the ONS classification scheme. Groups that were mixed White and Black African, Black Caribbean, or Asian were combined with Black African, Black Caribbean, and South Asian groups respectively due to smaller sample sizes.

While we included all ethnicity groups to inform our models, “other” groups likely include very heterogeneous populations with different characteristics and therefore may be uninformative or lead to misleading conclusions. We focus our discussion here on five more specific ethnicity groups with larger sample sizes: Black African, Black Caribbean, South Asian, White British, and White Irish groups.

In addition to ethnicity, we included in our models the following baseline demographic variables: age at diagnosis, gender, and neighbourhood-level deprivation. Deprivation was measured at the 2011 lower layer super output area (LSOA) boundaries, which are areas with populations of around 1700 residents [15]. Deprivation is reported as a continuous variable between 0 and 60, with higher numbers indicating more deprived areas.

We also included clinical variables related to dementia and other comorbidities. The Mini-Mental State Examination (MMSE) score was used as a measure of cognitive function/dementia severity within a 6-month period of the index diagnosis [16].

Two items from the Health of the Nations Outcome Scores (“Problems with activities of daily living” and “Physical illness or disability problems”) were used as measures of physical health comorbidities within a 6-month period of the index diagnosis [17]. Scores between 2 and 4 on these items were used as indicators of “some problems” with physical health.

Mental health comorbidities were also ascertained by searching the record for ICD-10 diagnoses of F10–F19 (mental and behavioural disorders due to psychoactive substance use, including alcohol, opioids, cannabinoids, sedatives, hypnotics, cocaine, stimulants, hallucinogens, tobacco, volatile solvents, and multiple drug use), F20–29 (schizophrenia, schizotypal, and delusional disorders), and F32–F33 (depressive episodes and recurrent depressive disorders) diagnosed on or before the index date. We also considered a score between 2 and 4 (“some problems”) on the HoNOS items “Problem drinking or drug-taking” and “Problems with depressed mood” as indicators of comorbid substance use disorders and depression respectively.

### Statistical methods

Data was cleaned in R version 4.0.5 and analysed in Stata 15 [18, 19].

#### Standardised mortality ratios

Standardised mortality ratios (SMR) and 95% CIs were calculated to compare death rates in our cohort to the number of deaths that would be expected based on the overall population in England and Wales, indirectly standardised by age and gender. SMRs were calculated for each of the five ethnicity groups of focus. Deaths reported in 2012 from the ONS were used, as this was the midpoint of our study follow-up period [20]. The overall population estimate was obtained from the ONS midyear 2012 report [21]. Age in our cohort was calculated as the patient’s age at the 31 December 2012 midpoint. This was aggregated into 5-year bands from ages 65–69, 70–74, 75–79, 80–84, and 85+ (excluding patients under 65 at the midpoint).

Because our cohort had a 10-year observation period but deaths in the standard population were for 1 year, we calculated weights by taking the mean observation period within each age and sex band in our cohort. These weights were then multiplied by number of deaths in each corresponding band in the reference/standard population, providing an estimate of expected deaths over the observation period.

#### Survival analysis

Kaplan-Meier survival curves were graphed for each ethnicity group using R packages survival and survminer [22, 23]. Log-rank tests were used to test for differences between curves.

Cox regression was used to compare hazard ratios for each minority ethnic group versus the White British group. Age at diagnosis, gender, deprivation score, and MMSE were added to the Cox regression models progressively based on prior literature on their association with mortality and ethnicity in dementia [9, 24]. For the association of age with death, a quadratic term for age produced a better fit for the model based on likelihood ratio tests and Bayesian information criteria. Two additional models were fitted to assess prior physical health comorbidities and prior mental health comorbidities. These models were also compared using likelihood-ratio tests.

For all models, the proportional hazards assumption was checked by testing for a non-zero slope in the Schoenfeld residuals over time. Variables violating the proportional hazards assumption were included in the model as time-varying covariates, and an interaction was fitted by multiplying these by time at risk. We additionally assessed interactions between ethnicity and other variables using likelihood ratio tests but decided not to include these in the final models.

Missing data was imputed using multiple imputation by chained equations, using ten imputations (MICE). The “Cognitive problems” HoNOS was additionally added as an auxiliary variable in the imputation regression model, as it was particularly correlated with MMSE, which had the most missing data (Pearson’s *r* = −0.46).

We additionally stratified by dementia subtype to examine whether estimates differed by subtype, as well as by age to further test whether estimates differed by age group (particularly as minority ethnic individuals tend to be younger at diagnosis).

#### Sensitivity analyses

We performed additional sensitivity analyses using a model including ethnicity, age, gender, deprivation, and MMSE score covariates to examine the robustness of our findings.

To explore whether differences in hazard ratios varied over time, we calculated hazard ratios for the cohort at progressively longer follow-up periods (1, 3, and 5 years post-diagnosis).

One hypothesis that has been proposed to explain previous findings of lower mortality in minority ethnic older adults has been that older adults born in another country may emigrate back to their country of birth when they become older and die there, leading to a numerator-denominator mismatch [12]. In order to account for this in our study, we used a competing risks regression model, which modifies the Cox regression model to include the possibility that participants may experience a “competing” event (emigration) which precludes them from experiencing the event of interest (death) [25]. Patients who were de-registered from the NHS during the follow-up period were considered to have emigrated out of the country in our model. The date at which the patient was de-registered was used as the censor date for the competing risk.

We also used a competing risks model to explore whether deaths from unnatural or external causes might explain any differences in survival, modelling unnatural-cause deaths as a competing risk for natural-cause death. The underlying cause of death from death certificates was ICD-10 coded and grouped into natural causes (A00-R99), unnatural causes (U509, V01-Y89), and not elsewhere classified (R00-R99) [26].

As minority ethnic individuals tend to be diagnosed at younger ages with dementia, it is possible that many may be diagnosed younger than 65 years of age. We also compared our model estimates when the cohort was expanded to include anyone over the age of 50.

A protocol for this analysis was pre-registered on Open Science Framework (osf.io).

## Results

### Demographic features

Fourteen thousand four hundred ninety-three patients aged 65 years and over had an index diagnosis of dementia between 01 January 2008 and 31 December 2017 and contributed 40,803.4 person-years at risk. The average length of follow-up from diagnosis was 2.8 years (SD 2.2) and median was 2.3 years (IQR: 25th percentile: 1.0 year, 75th percentile: 4.2 years). Maximum follow-up time for the full cohort was 10 years. 61.3% of patients were female, and average age at index diagnosis was 80 years. The majority (62.1%) of patients were recorded as White British, and the Black Caribbean group was the second-largest ethnicity group (11.9%). 2.7% of patients had missing ethnicity data. Dementia in Alzheimer’s disease was the most common subtype (39.9%), with mixed dementia (22.7%) as the second most common.

A total of 7611 (52.5%) patients died during follow-up, and median time to death was 2.02 years after index diagnosis (mean 2.47, SD 2.00).

Demographics of the overall cohort and for those who did and did not die during follow-up are shown in Table 1. Demographic characteristics and comorbidities for each ethnic group are tabulated in Supplementary Table 1. Ethnicity data was missing for 389 patients (2.7%). Those with missing ethnicity had on average shorter survival times (log rank test *p* <0.001).

**Table 1:** Cohort demographics by death status at the end of the follow-up period

	Alive (*N* = 6882)	Died(*N* = 7611)	Overall (*N* = 14,493)
**Ethnicity**			
Black African	282 (4.1%)	128 (1.7%)	410 (2.8%)
Black Caribbean	1091 (15.9%)	689 (9.1%)	1780 (12.3%)
South Asian	271 (3.9%)	177 (2.3%)	448 (3.1%)
White British	3715 (54.0%)	5278 (69.3%)	8993 (62.1%)
White Irish	326 (4.7%)	348 (4.6%)	674 (4.7%)
Any other Asian background	158 (2.3%)	118 (1.6%)	276 (1.9%)
Any other Black background	117 (1.7%)	66 (0.9%)	183 (1.3%)
Any other ethnic group or any other mixed background^a^	302 (4.4%)	183 (2.4%)	485 (3.3%)
Any other White background	460 (6.7%)	395 (5.2%)	855 (5.9%)
Missing	160 (2.3%)	229 (3.0%)	389 (2.7%)
**Age at diagnosis**			
Mean (SD)	80.4 (7.00)	83.5 (7.00)	82.0 (7.18)
**Gender**			
Female	4380 (63.6%)	4498 (59.1%)	8878 (61.3%)
Male	2502 (36.4%)	3111 (40.9%)	5613 (38.7%)
**MMSE score within 6 months of index diagnosis**			
Mean (SD)	19.4 (6.20)	17.6 (6.27)	18.5 (6.30)
**Index of multiple deprivation score (based on neighbourhood)**^**b**^			
Mean (SD)	26.3 (11.5)	26.6 (11.6)	26.5 (11.6)
**Subtype**			
Lewy body dementia	287 (4.2%)	357 (4.7%)	644 (4.4%)
Mixed dementia	1547 (22.5%)	1740 (22.9%)	3287 (22.7%)
Other or unspecified dementia	689 (10.0%)	1365 (17.9%)	2054 (14.2%)
Alzheimer’s disease	3332 (48.4%)	2457 (32.3%)	5789 (39.9%)
Vascular dementia	1027 (14.9%)	1692 (22.2%)	2719 (18.8%)

Missing data: 11 patients were missing data on age, 2 were missing data on gender, 3472 on MMSE score, and 85 on index of multiple deprivation score

^a^Combined in this table to suppress small numbers

^b^Higher numbers indicate areas with higher deprivation

### Standardised mortality ratios

Compared to the population in England and Wales in 2012, our overall cohort had an elevated age- and gender-standardised death rate (SMR: 2.48, 95% CI 2.43–2.54, *n* = 14,260). In all ethnicity groups, death rates were elevated for all-cause mortality compared to the standard population. The SMR in the Black African group was 2.74 (95% CI 2.29–3.26), in the Black Caribbean group was 2.06 (95% CI 1.91–2.22), in the South Asian group 2.44 (95% CI 2.10–2.83), in the White Irish group 2.74 (95% CI 2.46–3.05), and in the White British group 2.67 (95% CI 2.60–2.74).

### Survival analysis

**Fig. 1:**
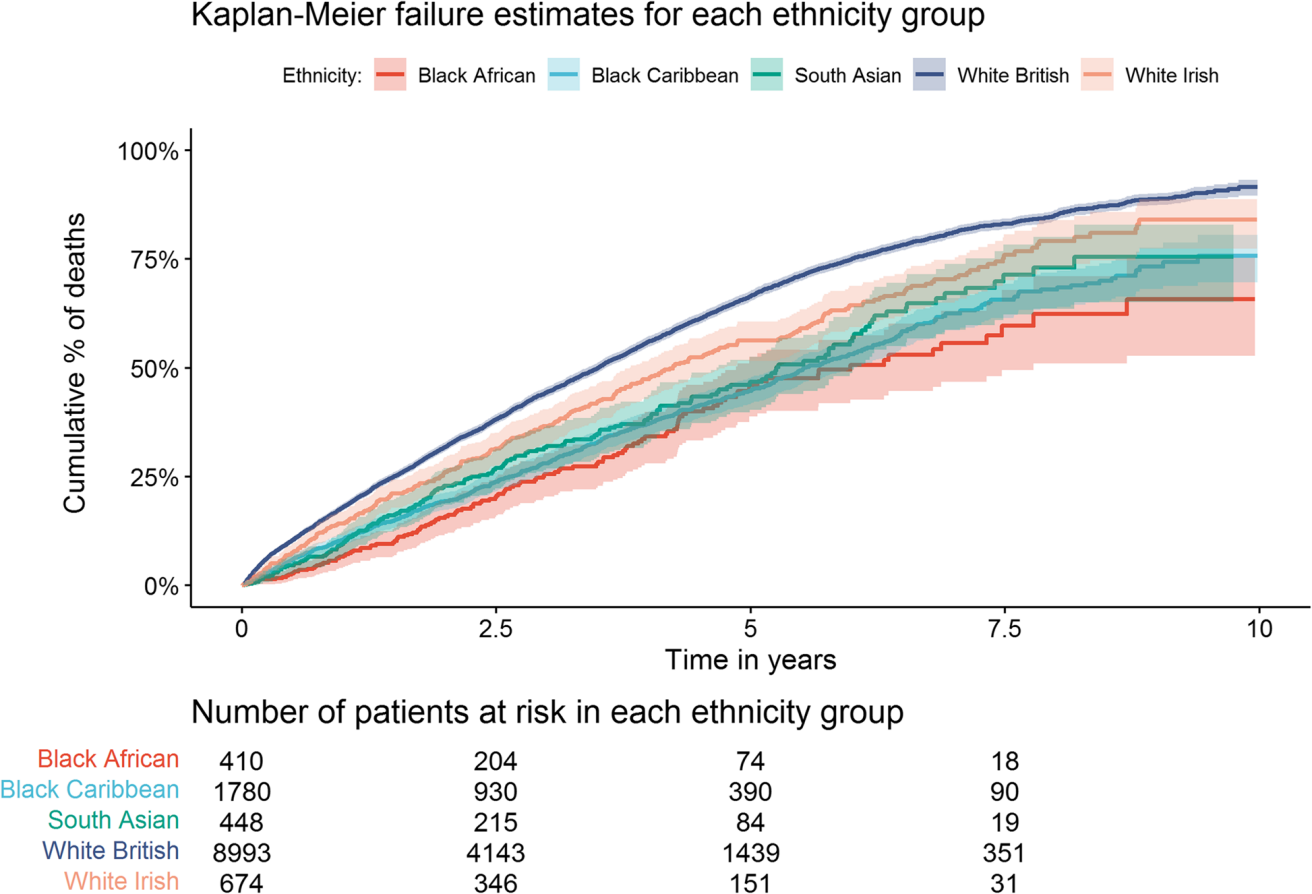
Kaplan-Meier failure curves comparing deaths in each ethnicity group

Figure 1 shows failure curves comparing the five ethnicity groups (White British, White Irish, Black Caribbean, Black African, South Asian). Log-rank tests comparing survival curves of each minority ethnic group to the White British comparison group indicated unequal survival functions (*p* <0.01).

Results for each model in the survival analysis are displayed in Table 2. Without controlling for other factors, all other ethnicity groups had a lower hazard of death compared to the White British group. For the Black Caribbean and Black African groups, the unadjusted hazard ratio for death was about half that of the White British group (HR for the Black Caribbean group: 0.57 (95% CI: 0.53–0.62), for the Black African group: 0.48 (95% CI: 0.40–0.57)). In the South Asian group, the hazard was 0.62 (95% CI: 0.53–0.72) times the hazard in the White British group, and in the White Irish group hazard was 0.77 (95% CI: 0.69–0.86) times the hazard in the White British group.

**Table 2:** Hazard ratios comparing survival in minority ethnic groups to the White British group for unadjusted and adjusted Cox regression models

Ethnicity	***n*** (%)	Model 1—unadjusted	Model 2—adjusted for age, gender	Model 3—adjusted for age, gender, MMSE, deprivation	Model 4—model 3 + mental health comorbidities	Model 5—model 3 + physical health comorbidities	Model 3 with competing risk of leaving NHS (***n*** = 10,766)^**a**^
**White British**	8993 (63.76%)	Ref	Ref	Ref	Ref	Ref	Ref
**Black African**	410 (2.91%)	0.48 (0.40–0.57)	0.68 (0.57–0.81)	0.60 (0.50–0.71)	0.60 (0.50–0.71)	0.56 (0.47–0.68)	0.64 (0.52–0.79)
**Black Caribbean**	1780 (12.62%)	0.57 (0.53–0.62)	0.64 (0.59–0.69)	0.57 (0.52–0.61)	0.57 (0.52–0.61)	0.56 (0.51–0.60)	0.55 (0.50–0.60)
**South Asian**	448 (3.18%)	0.62 (0.53–0.72)	0.74 (0.64–0.86)	0.70 (0.60–0.82)	0.69 (0.60–0.81)	0.71 (0.61–0.83)	0.68 (0.58–0.81)
**White Irish**	674 (4.78%)	0.77 (0.69–0.86)	0.89 (0.80–0.99)	0.84 (0.75–0.93)	0.83 (0.74–0.93)	0.82 (0.73–0.91)	0.86 (0.76–0.98)
**Any other Asian background**	276 (1.96%)	0.69 (0.57–0.83)	0.82 (0.69–0.99)	0.75 (0.62–0.90)	0.76 (0.63–0.91)	0.75 (0.63–0.90)	0.73 (0.58–0.92)
**Any other Black background**	183 (1.3%)	0.57 (0.45–0.72)	0.69 (0.54–0.88)	0.63 (0.49–0.80)	0.62 (0.49–0.79)	0.62 (0.49–0.79)	0.57 (0.42–0.77)
**Any other ethnic group**	464 (3.29%)	0.68 (0.59–0.79)	0.76 (0.65–0.88)	0.70 (0.61–0.82)	0.70 (0.60–0.81)	0.71 (0.61–0.82)	0.62 (0.52–0.75)
**Any other mixed background**	21 (0.15%)	0.52 (0.25–1.09)	0.63 (0.30–1.32)	0.61 (0.29–1.30)	0.63 (0.30–1.32)	0.64 (0.30–1.35)	0.42 (0.16–1.10)
**Any other White background**	855 (6.06%)	0.69 (0.62–0.76)	0.73 (0.65–0.81)	0.66 (0.59–0.73)	0.65 (0.59–0.72)	0.65 (0.59–0.73)	0.64 (0.56–0.72)

For models including age, MMSE, mental health comorbidities (prior depression, prior schizophrenia/schizotypal/delusional disorders), and physical health comorbidities (HoNOS physical illness subscale, and HoNOS activities of daily living subscale), each of these variables was treated as time-varying covariates and an interaction with time was included in the model

*SD* Standard deviation

^a^Complete case analysis used

As some minority ethnic groups had a younger average age at first diagnosis, a model including squared age at diagnosis (as a time-varying covariate), gender, and ethnicity attenuated this in all groups: the HR for the Black Caribbean group compared to the White British group increased from 0.57 (95% CI: 0.53–0.62) to 0.64 (95% CI: 0.59–0.69), in the Black African group from 0.48 (95% CI: 0.40–0.57) to 0.68 (95% CI: 0.57–0.81), in the South Asian group from 0.62 (95% CI: 0.53–0.72) to 0.74 (95% CI: 0.64–0.86), and in the White Irish group from 0.77 (95% CI: 0.69–0.86) to 0.89 (95% CI: 0.80–0.99).

A third model was fitted adding neighbourhood-level deprivation scores and MMSE scores within 6 months of the index diagnosis. The HRs remained roughly similar in all groups or decreased slightly closer to the unadjusted model: in the Black Caribbean group the HR became 0.57 (95% CI: 0.52–0.61), in the Black African group 0.60 (95% CI: 0.50–0.71), in the South Asian group 0.70 (95% CI: 0.60–0.82), in the White Irish group 0.84 (95% CI: 0.75–0.93). The hazard of death was also elevated for patients who were male, older at diagnosis, lived in more deprived areas, and who had lower index MMSE scores (see Supplementary Table 2 for full covariate estimates).

A fourth and fifth model also included indicators of prior mental health problems (substance use, depression, and schizophrenia and related disorders) and physical health problems close to the index date (based on HoNOS physical illness and activities of daily living subscales). While indicators of depression, schizophrenia/schizotypal/delusional disorder, physical illness, and difficulties with activities of daily living were associated with a higher hazard ratio for death, adding these covariates to the model did not substantially change estimates in any of the ethnicity groups. Likelihood ratio tests comparing each successive model as variables were added were all *p*<0.0001. The complete case analysis produced similar results to the imputed model for the Black African, Black Caribbean, South Asian, and White Irish groups compared to White British (see Supplementary Table 3).

When stratifying the results by subtype, there were too few individuals diagnosed with Lewy body dementia in most ethnicity groups, resulting in low power. However, the hazard of death remained lower compared to White British groups across all subtypes for the Black African and Black Caribbean groups. In the South Asian group compared to the White British group, there was no evidence of difference in mortality in mixed and vascular dementias. In the White Irish group, there was also no evidence of difference compared to White British groups with mixed and Alzheimer’s subtypes (see Table 3).

**Table 3:** Hazard ratios comparing survival in minority ethnic groups to the White British group, stratified by dementia subtype

Ethnicity	Lewy body dementia (***n*** = 644)	Mixed dementia (***n*** = 3287)	Other/unspecified (***n*** = 2054)	Alzheimer’s disease (***n*** = 5789)	Vascular dementia (***n*** = 2719)
**Black African**	**0.77 (0.34–1.77)**	**0.67 (0.48–0.94)**	**0.39 (0.24–0.61)**	**0.55 (0.36–0.84)**	**0.68 (0.49–0.93)**
**Black Caribbean**	**0.46 (0.31–0.70)**	**0.53 (0.45–0.63)**	**0.59 (0.48–0.73)**	**0.55 (0.47–0.65)**	**0.62 (0.54–0.73)**
**South Asian**	**0.55 (0.28–1.08)**	**0.73 (0.52–1.01)**	**0.68 (0.47–0.98)**	**0.71 (0.55–0.91)**	**0.80 (0.58–1.10)**
**White Irish**	**0.74 (0.47–1.17)**	**0.82 (0.65–1.03)**	**0.76 (0.59–0.98)**	**0.94 (0.77–1.14)**	**0.76 (0.60–0.96)**
Any other Asian background	0.55 (0.20–1.48)	0.71 (0.48–1.06)	0.96 (0.64–1.44)	0.54 (0.38–0.78)	1.18 (0.83–1.68)
Any other Black background	0.54 (0.22–1.31)	0.69 (0.38–1.27)	0.54 (0.28–1.01)	0.7 (0.45–1.08)	0.54 (0.34–0.84)
Any other ethnic group	0.61 (0.28–1.31)	0.86 (0.64–1.15)	0.81 (0.56–1.17)	0.69 (0.53–0.89)	0.63 (0.44–0.90)
Any other mixed background			1.50 (0.48–4.74)	0.57 (0.14–2.31)	0.68 (0.17–2.76)
Any other White background	0.72 (0.44–1.20)	0.77 (0.62–0.95)	0.67 (0.52–0.86)	0.57 (0.47–0.69)	0.73 (0.59–0.91)

All models adjust for age at diagnosis (squared), gender, MMSE score near index date, and neighbourhood-level deprivation

Despite younger average age at diagnosis, hazard of survival remained lower across all age strata for the Black Caribbean, Black African, and South Asian groups as compared with the White British group. For 65- to 75-year-olds and 75- to 85-year-olds, the difference in survival between the White Irish and White British group became nonsignificant (see Table 4).

**Table 4:** Hazard ratios comparing survival in minority ethnic groups to the White British group, stratified by age at diagnosis

Ethnicity	65–75 years old (***n*** = 1866)	75–85 years old (***n*** = 5006)	85–95 years old (***n*** = 3666)
**Black African**	**0.65 (0.47–0.92)**	**0.59 (0.44–0.79)**	**0.55 (0.31–0.96)**
**Black Caribbean**	**0.60 (0.47–0.75)**	**0.54 (0.48–0.62)**	**0.51 (0.43–0.60)**
**South Asian**	**0.54 (0.36–0.80)**	**0.71 (0.56–0.91)**	**0.65 (0.46–0.92)**
**White Irish**	**0.79 (0.59–1.06)**	**0.88 (0.73–1.05)**	**0.78 (0.63–0.97)**
Any other Asian background	0.77 (0.44–1.33)	0.69 (0.51–0.93)	0.82 (0.53–1.28)
Any other Black background	0.92 (0.54–1.57)	0.39 (0.24–0.63)	0.54 (0.30–0.95)
Any other ethnic group	0.61 (0.36–1.02)	0.50 (0.38–0.67)	0.73 (0.55–0.97)
Any other mixed background	0.22 (0.03–1.58)	0.55 (0.08–3.90)	0.50 (0.12–1.99)
Any other White background	0.61 (0.42–0.88)	0.62 (0.52–0.74)	0.63 (0.52–0.77)

All models adjust for gender, MMSE score near index date, and neighbourhood-level deprivation. Age bands are inclusive of the upper bound and exclusive of the lower bound (with the exception of 65 years old)

There were 7223 deaths from natural causes, 120 deaths from unnatural causes, and 268 deaths with an unknown or “not elsewhere classified” cause. Across all minority ethnic groups compared to White British group, the hazard of death from natural causes, accounting for competing risk of dying of unnatural causes, was similar to the overall hazard ratio of death.

### Sensitivity analyses

We additionally performed sensitivity analyses using the third model which included ethnicity, age, gender, index of multiple deprivation, and index MMSE score. These are reported in Supplementary Table 3.

Although in our initial models we accounted for violations of proportional hazards assumptions by adding interactions with time, we also examined how hazard ratios compare over shorter follow-up periods. For Black Caribbean, Black African, and South Asian groups, the hazard ratio for mortality at 1, 3, and 5 years remained lower than in the White British reference group. HRs remained similar across all follow-up period lengths, and there was no clear trend when cutting off the maximum follow-up time at 1, 3, 5, and 10 years.

During the follow-up period, 169 patients left the NHS. In a competing risks regression model accounting for the competing risk of leaving the NHS, the hazard ratio for all four minority ethnic groups compared to the White British group changed little if at all compared to the model without competing risks.

## Discussion

Across all ethnicity groups in our cohort of older adults with dementia in South London, mortality was elevated by at least double the age- and gender-standardised reference population. Within our cohort of people living with dementia, we found that Black Caribbean, Black African, White Irish, and South Asian groups may have a lower risk of death compared to White British individuals with dementia, even after accounting for age at diagnosis, gender, neighbourhood-level deprivation, cognitive functioning at baseline, and mental and physical comorbidities. These differences in risk were robust to a range of sensitivity analyses, including shortening follow-up to 1-, 3-, and 5-year periods as well as accounting for potential numerator-denominator mismatch due to individuals moving away during the follow-up period. These results were mostly consistent across dementia subtypes, though some differences were not detected in certain subtypes, likely due to smaller sample sizes in these groups. Results were also similar when stratifying by 10-year age bands, though differences were not detected for the White Irish group in younger age bands. This may be due to reduced power in this group or may indicate that some differences in survival may be driven by longer survival amongst the older White Irish individuals.

These findings are similar to previous findings which suggested that non-White older adults with dementia had a lower risk of death versus a White comparison group with dementia, although specific minority ethnic groups were not assessed [27]. Another study in Camden and Islington in London also found that Asian dementia patients had half the mortality risk of their White British counterparts and that White Irish and mixed minority ethnic groups also had a lower risk of mortality [28].

Within UK populations with severe mental illness, depression, delirium, and the general population, minority ethnic groups also seem to live longer compared to White British groups [29–33]. However, the studies relating to samples with mental disorders (severe mental illness and depression) also indicated a two to threefold increase in age/sex-standardised mortality ratios, compared to the general population across all ethnicity groups [29, 30]. In the severe mental illness population, longer survival may be associated with living in areas with higher own-group ethnic density, as similar mortality risks were found for minority ethnic individuals living in areas of lower own-ethnic density [29]. Future research may investigate whether there is a relationship between ethnic density and survival for individuals with dementia.

In the general population, findings of lower mortality in minority ethnic groups have been hypothesised to be a result of immigration patterns and the so-called healthy migrant hypothesis. When studies separate UK-born and non-UK-born individuals in the same minority ethnic group, the effect of lower mortality versus comparison groups only holds for non-UK-born minority ethnic individuals. However, we were unable to confirm whether patients in our study were born in the UK or had immigrated [31, 32].

These findings of longer survival in dementia are crucial to care planning for individuals with dementia and their families, as prolonged duration of disease may increase emotional and financial stress and can impact carer’s mental and physical health and exacerbate family conflict [34]. Increased carer burden is also associated with a higher risk of institutionalisation for the person living with dementia [34, 35]—which in turn is associated with shorter survival [36]. Longer survival may also result in increased expenses related to medical or residential care for families. Minority ethnic individuals additionally face barriers to accessing care, including discrimination and stigma, which may make it more difficult to get support over a longer period with the disease [5, 7, 8].

### Strengths and limitations

Ethnicity was missing for less than 3% of the sample, indicating relatively complete recording in the clinical record. However, we did not have access to other variables such as education and individual-level socioeconomic status which may confound the association between ethnicity and survival, although we were able to adjust for area-level deprivation.

In our models, we chose to include some ethnicity groups such as “any other Black background” and “any other White background”, which may be particularly heterogeneous, and therefore findings for these groups may be less informative. While these groups also had a lower risk for mortality compared to the White British group (apart from the “any other mixed background” group, which was small and likely underpowered), these estimates are more difficult to interpret as they may be combining disparate groups with different experiences of migration and settlement and different health risks.

Previous literature suggests that certain minority ethnic groups may present to services later after the onset of dementia, based on qualitative research indicating that minority ethnic individuals may be more likely to make first contact with dementia services via emergency or crisis services and from findings of higher cognitive impairment scores at diagnosis [7, 9, 37, 38]. The effect of longer survival may thus be even more pronounced if measuring from onset of dementia symptoms. Because our analysis was limited to data available through secondary care routine health records, we assumed a first diagnosis date to be the first instance a dementia diagnosis was recorded by a secondary care physician, but onset of dementia may have occurred earlier or may have been discussed at a primary care level prior to it being recorded in the health records. Average MMSE scores across minority ethnic groups in our cohort were lower than in the White British group, suggesting that diagnosis may have been later on average in these groups in our cohort as well. However, the use of data from free text fields to identify dementia diagnosis may provide additional sensitivity in identifying people living with dementia prior to a structured ICD-10 code diagnosis.

A further limitation to using electronic health record data to identify people living with dementia is that only individuals with a formal diagnosis or who are accessing formal health services are represented. In the UK in 2017 and 2018 (around the end of the study period), it was estimated that around a third of dementia cases were never formally diagnosed [39]. Minority ethnic groups in particular may face additional barriers to receiving a formal diagnosis; a previous study found that Black men in particular may be less likely to receive formal diagnoses based on comparisons in incidence rates calculated using primary care record data versus using data from community cohort studies [2]. Lower incidence of dementia in health records was also reported in South Asian groups, though lack of community-based research makes it difficult to determine whether this is due to underdiagnosis [2]. It is then possible that survival rates may differ for individuals with undiagnosed dementia, disproportionately affecting minority ethnic groups. For example, mortality risk might be higher in undiagnosed individuals if the lack of diagnosis prevents individuals from accessing treatments which slow the rate of cognitive decline. Ethnic differences in mortality risk might also be overestimated if minority ethnic individuals with faster rates of cognitive decline are missed from clinical records if they die before receiving a formal diagnosis.

Our analysis focused on a large mental health trust in South London, which may not be generalisable to England as a whole, although may be typical of other urban and ethnically diverse areas across the UK. In areas with different accessibility of care and community support, the timeliness of diagnosis and quality of care might impact survival estimates for minority ethnic individuals.

## Conclusions

Future research might explore possible explanations for these differences in survival, including community-level factors such as ethnic density and accessibility of care as well as hypotheses around data quality and migration patterns. Equally, research on how longer survival may affect individuals with dementia and their families will be important for ensuring that carers from minority ethnic groups are supported adequately.

## Supplementary Information

**Additional file 1: Supplementary Figure 1.** Algorithm for determining dementia subtype. **Supplementary Table 1.** Cohort demographics by ethnicity group. **Supplementary Table 2.** Model estimates including additional covariates. **Supplementary Table 3.** Additional sensitivity analyses using Model 3.

## Data Availability

Data are owned by a third party, Maudsley Biomedical Research Centre (BRC) Clinical Records Interactive Search (CRIS) tool, which provides access to anonymised data derived from SLaM electronic medical records. These data can only be accessed by permitted individuals from within a secure firewall (i.e. the data cannot be sent elsewhere), in the same manner as the authors. For more information, please contact cris.administrator@slam.nhs.uk.
