# Supplementary material for: Ethnicity and survival after a dementia diagnosis: a retrospective cohort study using electronic health record data
Source: Alzheimers Res Ther. 2023 Mar 29;15:67. doi: 10.1186/s13195-022-01135-z (PMC10052806; doi:10.1186/s13195-022-01135-z)
Supplement: Supplementary file 1 — Additional file 1: Supplementary Figure 1. Algorithm for determining dementia subtype. Supplementary Table 1. Cohort demographics by ethnicity group. Supplementary Table 2. Model estimates including additional covariates. Supplementary Table 3. Additional sensitivity analyses using Model 3. [file 13195_2022_1135_MOESM1_ESM.docx]

## Supplementary Material

### Supplementary Figure 1- Algorithm for determining dementia subtype

Total cohort

(N = 14,493)

N = 4,773

N = 10,582

Mixed dementia

(n = 3,287)

Lewy body dementia

(n = 644)

N = 13,849

Alzheimer’s Disease

(n = 5,789)

Vascular dementia

(n = 2,719)

Ever had a diagnosis of vascular dementia

Ever had a diagnosis of Alzheimer’s Disease

Both Alzheimer’s and vascular diagnoses in the EHR, or a diagnosis of mixed type dementia (F00.2)

Ever had a diagnosis of Lewy body dementia

Other or unspecified dementias

(n = 2,054)

### Supplementary Table 1- Cohort demographics by ethnicity group

|  | **Black African**  **(N=410)** | **Black Caribbean**  **(N=1,780)** | **South Asian**  **(N=448)** | **White British**  **(N=8,993)** | **White Irish**  **(N=674)** | **Any other Asian background**  **(N=276)** | **Any other Black background**  **(N=183)** | **Any other ethnic group**  **(N=464)** | **Any other mixed background**†  **(N=21)** | **Any other White background**  **(N=855)** | **Missing**  **(N=389)** | **Overall**  **(N=14,493)** |
| --- | --- | --- | --- | --- | --- | --- | --- | --- | --- | --- | --- | --- |
|  |  |  |  |  |  |  |  |  |  |  |  |  |
| **Gender** |  |  |  |  |  |  |  |  |  |  |  |  |
| Female | 225 (54.9%) | 1,015 (57.0%) | 239 (53.3%) | 5,652 (62.8%) | 406 (60.2%) | 156 (56.5%) | 102 (55.7%) | 275 (59.3%) | - | 547 (64.0%) | 252 (64.8%) | 8,878 (61.3%) |
| Male | 185 (45.1%) | 764 (42.9%) | 209 (46.7%) | 3,341 (37.2%) | 268 (39.8%) | 119 (43.1%) | 81 (44.3%) | 189 (40.7%) | - | 308 (36.0%) | 137 (35.2%) | 5,613 (38.7%) |
| **Age at diagnosis** |  |  |  |  |  |  |  |  |  |  |  |  |
| Mean (SD) | 76.5 (6.6) | 80.6 (6.4) | 79.4 (6.8) | 82.9 (7.2) | 80.2 (7.0) | 80.2 (6.8) | 79.6 (7.0) | 81.3 (7.2) | 78.3 (7.7) | 81.7 (7.0) | 83.6 (7.5) | 82.0 (7.2) |
| **Age at diagnosis (5-year age bands)*** |  |  |  |  |  |  |  |  |  |  |  |  |
| 65-70 | 68 (16.6%) | 96 (5.4%) | 50 (11.2%) | 443 (4.9%) | 51 (7.6%) | 20 (7.2%) | 18 (9.8%) | 39 (8.4%) | - | 57 (6.7%) | 21 (5.4%) | 866 (6.0%) |
| 70-75 | 114 (27.8%) | 264 (14.8%) | 72 (16.1%) | 913 (10.2%) | 111 (16.5%) | 37 (13.4%) | 35 (19.1%) | 49 (10.6%) | - | 90 (10.5%) | 36 (9.3%) | 1,727 (11.9%) |
| 75-80 | 109 (26.6%) | 448 (25.2%) | 116 (25.9%) | 1584 (17.6%) | 161 (23.9%) | 81 (29.3%) | 42 (23.0%) | 104 (22.4%) | - | 182 (21.3%) | 52 (13.4%) | 2,882 (19.9%) |
| 80-85 | 69 (16.8%) | 512 (28.8%) | 114 (25.4%) | 2257 (25.1%) | 163 (24.2%) | 69 (25.0%) | 44 (24.0%) | 120 (25.9%) | - | 234 (27.4%) | 98 (25.2%) | 3,683 (25.4%) |
| 85-90 | *Ages 85+*:  50 (12.2%) † | 343 (19.3%) | 68 (15.2%) | 2396 (26.6%) | 135 (20.0%) | 48 (17.4%) | 32 (17.5%) | 104 (22.4%) | - | 203 (23.7%) | 89 (22.9%) | 3,464 (23.9%) |
| 90+ |  | 117 (6.6%) | 28 (6.3%) | 1399 (15.6%) | 53 (7.9%) | 21 (7.6%) | 12 (6.6%) | 48 (10.3%) | - | 89 (10.4%) | 83 (21.3%) | 1,860 (12.8%) |
| **MMSE** |  |  |  |  |  |  |  |  |  |  |  |  |
| Mean (SD) | 17.7 (6.8) | 17.6 (6.2) | 18.2 (6.7) | 18.9 (6.2) | 18.3 (6.1) | 17.3 (6.9) | 18.0 (6.8) | 17.7 (6.6) | 19.5 (5.5) | 16.9 (6.3) | 17.8 (6.7) | 18.5 (6.3) |
| **Subtype** |  |  |  |  |  |  |  |  |  |  |  |  |
| Lewy body | 14 (3.4%) | 61 (3.4%) | 21 (4.7%) | 426 (4.7%) | 35 (5.2%) | 13 (4.7%) | 12 (6.6%) | 17 (3.7%) | - | 31 (3.6%) | 14 (3.6%) | 644 (4.4%) |
| Mixed | 111 (27.1%) | 451 (25.3%) | 103 (23.0%) | 2,013 (22.4%) | 152 (22.6%) | 57 (20.7%) | 34 (18.6%) | 108 (23.3%) | - | 207 (24.2%) | 48 (12.3%) | 3,287 (22.7%) |
| Other/ unspecified | 50 (12.2%) | 186 (10.4%) | 57 (12.7%) | 1,354 (15.1%) | 107 (15.9%) | 39 (14.1%) | 22 (12.0%) | 51 (11.0%) | - | 108 (12.6%) | 76 (19.5%) | 2,054 (14.2%) |
| Pure Alzheimer’s | 136 (33.2%) | 675 (37.9%) | 195 (43.5%) | 3,622 (40.3%) | 250 (37.1%) | 115 (41.7%) | 71 (38.8%) | 213 (45.9%) | - | 353 (41.3%) | 150 (38.6%) | 5,789 (39.9%) |
| Pure vascular | 99 (24.1%) | 407 (22.9%) | 72 (16.1%) | 1,578 (17.5%) | 130 (19.3%) | 52 (18.8%) | 44 (24.0%) | 75 (16.2%) | - | 156 (18.2%) | 101 (26.0%) | 2,719 (18.8%) |
| **Index of multiple deprivation score** |  |  |  |  |  |  |  |  |  |  |  |  |
| Mean (SD) | 31.5 (9.0) | 30.5 (10.1) | 24.9 (10.1) | 25.3 (11.9) | 28.8 (11.0) | 26.9 (11.2) | 29.1 (10.3) | 25.7 (11.7) | 28.1 (12.5) | 27.4 (10.5) | 25.2 (11.5) | 26.5 (11.6) |
| **Prior substance use problems** |  |  |  |  |  |  |  |  |  |  |  |  |
| N (%) | † | 72 (4.0%) | † | 314 (3.5%) | 61 (9.1%) | 11 (4.0%) |  | 15 (3.2%) | - | 26 (3.0%) |  | 529 (3.7%) |
| **Prior depression** |  |  |  |  |  |  |  |  |  |  |  |  |
| N (%) | 78 (19.0%) | 290 (16.3%) | 88 (19.6%) | 1,525 (17.0%) | 154 (22.8%) | 55 (19.9%) | 33 (18.0%) | 92 (19.8%) | - | 187 (21.9%) | 32 (8.2%) | 2,537 (17.5%) |
| **Prior schizophrenia or related disorders** |  |  |  |  |  |  |  |  |  |  |  |  |
| N (%) | 21 (5.1%) | 136 (7.6%) | 24 (5.4%) | 247 (2.7%) | 34 (5.0%) | 17 (6.2%) | 11 (6.0%) | 12 (2.6%) | - | 45 (5.3%) |  | 553 (3.8%) |
| **Physical health problems** |  |  |  |  |  |  |  |  |  |  |  |  |
| N (%) | 230 (56.1%) | 959 (53.9%) | 217 (48.4%) | 4,589 (51.0%) | 361 (53.6%) | 144 (52.2%) | 88 (48.1%) | 227 (48.9%) | - | 457 (53.5%) | 176 (45.2%) | 7,458 (51.5%) |
| Missing | 32 (7.8%) | 147 (8.3%) | 40 (8.9%) | 833 (9.3%) | 58 (8.6%) | 28 (10.1%) | 20 (10.9%) | 48 (10.3%) | - | 82 (9.6%) | 111 (28.5%) | 1,401 (9.7%) |
| **Problems with activities of daily living** |  |  |  |  |  |  |  |  |  |  |  |  |
| N (%) | 244 (59.5%) | 1,016 (57.1%) | 212 (47.3%) | 4,948 (55.0%) | 396 (58.8%) | 157 (56.9%) | 103 (56.3%) | 243 (52.4%) | - | 467 (54.6%) | 182 (46.8%) | 7,978 (55.0%) |
| Missing | 35 (8.5%) | 148 (8.3%) | 40 (8.9%) | 858 (9.5%) | 58 (8.6%) | 28 (10.1%) | 21 (11.5%) | 49 (10.6%) | - | 84 (9.8%) | 111 (28.5%) | 1,434 (9.9%) |

† Further breakdown not reported due to suppression of small numbers

* Inclusive of higher age bound

### Supplementary Table 2- Model estimates including additional covariates

|  | n (%) | Model 1- Unadjusted | Model 2- Adjusted for age, gender | Model 3- Adjusted for age, gender, MMSE, deprivation | Model 4- Model 3 + mental health comorbidities | Model 5- Model 3 + physical health comorbidities | Model 3 with competing risk of leaving NHS  (n = 10,766) ‡ |
| --- | --- | --- | --- | --- | --- | --- | --- |
| *Ethnicity* |  |  |  |  |  |  |  |
| White British | 8,993 (63.76%) | Reference | Ref | Ref | Ref | Ref | Ref |
| Black African | 410 (2.91%) | 0.48 (0.40-0.57) | 0.68 (0.57-0.81) | 0.60 (0.50-0.71) | 0.60 (0.50-0.71) | 0.56 (0.47-0.68) | 0.64 (0.52-0.79) |
| Black Caribbean | 1,780 (12.62%) | 0.57 (0.53-0.62) | 0.64 (0.59-0.69) | 0.57 (0.52-0.61) | 0.57 (0.52-0.61) | 0.56 (0.51-0.60) | 0.55 (0.50-0.60) |
| South Asian | 448 (3.18%) | 0.62 (0.53-0.72) | 0.74 (0.64-0.86) | 0.70 (0.60-0.82) | 0.69 (0.60-0.81) | 0.71 (0.61-0.83) | 0.68 (0.58-0.81) |
| White Irish | 674 (4.78%) | 0.77 (0.69-0.86) | 0.89 (0.80-0.99) | 0.84 (0.75-0.93) | 0.83 (0.74-0.93) | 0.82 (0.73-0.91) | 0.86 (0.76-0.98) |
| Any other Asian background | 276 (1.96%) | 0.69 (0.57-0.83) | 0.82 (0.69-0.99) | 0.75 (0.62-0.90) | 0.76 (0.63-0.91) | 0.75 (0.63-0.90) | 0.73 (0.58-0.92) |
| Any other Black background | 183 (1.3%) | 0.57 (0.45-0.72) | 0.69 (0.54-0.88) | 0.63 (0.49-0.80) | 0.62 (0.49-0.79) | 0.62 (0.49-0.79) | 0.57 (0.42-0.77) |
| Any other ethnic group | 464 (3.29%) | 0.68 (0.59-0.79) | 0.76 (0.65-0.88) | 0.70 (0.61-0.82) | 0.70 (0.60-0.81) | 0.71 (0.61-0.82) | 0.62 (0.52-0.75) |
| Any other mixed background | 21 (0.15%) | 0.52 (0.25-1.09) | 0.63 (0.30-1.32) | 0.61 (0.29-1.30) | 0.63 (0.30-1.32) | 0.64 (0.30-1.35) | 0.42 (0.16-1.10) |
| Any other White background | 855 (6.06%) | 0.69 (0.62-0.76) | 0.73 (0.65-0.81) | 0.66 (0.59-0.73) | 0.65 (0.59-0.72) | 0.65 (0.59-0.73) | 0.64 (0.56-0.72) |
|  |  |  |  |  |  |  |  |
| *Covariates* |  |  |  |  |  |  |  |
| Age at diagnosis, squared | Mean: 82.0  SD: 7.18 |  | 1.00 (1.00-1.00) | 1.00 (1.00-1.00) | 1.00 (1.00-1.00) | 1.00 (1.00-1.00) | 1.00 (1.00-1.00) |
| Male gender | 5,613 (38.7%) |  | 1.48 (1.41-1.55) | 1.51 (1.44-1.58) | 1.52 (1.45-1.60) | 1.49 (1.42-1.56) | 1.55 (1.47-1.64) |
| Index of multiple deprivation | Mean: 26.5 SD: 11.6 |  |  | 1.01 (1.00-1.01) | 1.00 (1.00-1.01) | 1.00 (1.00-1.00) | 1.01 (1-1.01) |
| Index MMSE Score | Mean: 18.5  SD: 6.30 |  |  | 0.95 (0.95-0.96) | 0.95 (0.95-0.96) | 0.96 (0.96-0.97) | 0.95 (0.95-0.96) |
| Prior substance use disorders/problems | 529 (3.65%) |  |  |  | 1.04 (0.92-1.18) |  |  |
| Prior depression | 2,537 (17.5%) |  |  |  | 1.35 (1.23-1.48) |  |  |
| Prior schizophrenia/ schizotypal/ delusional disorders | 553 (3.8%) |  |  |  | 1.37 (1.13-1.67) |  |  |
| HoNOS Physical illness item† | 7,458 (57.0%) |  |  |  |  | 2.02 (1.84-2.22) |  |
| HoNOS Activities of daily living item† | 7,978 (61.1%) |  |  |  |  | 1.58 (1.44-1.74) |  |

For models including age^2^, MMSE, prior depression, prior schizophrenia/schizotypal/delusional disorders, HoNOS physical illness subscale, and HoNOS activities of daily living subscale, each of these variables were treated as time-varying covariates and an interaction with time was included in the model. Time interactions were 1.00 (1.00-1.00) because they were very small.

† for HoNOS scores, the hazard of having at least “some problems” (a score of 2 or higher) compared to little or no problems (a score of 0 or 1)

‡ complete case analysis used

### Supplementary Table 3- Additional sensitivity analyses using Model 3 (age, gender, deprivation, index MMSE)

| Ethnicity | Model 3 | Complete case analysis- Model 3  (n = 10,774) | Model 3 with one year follow-up  (n = 10,774) | Model 3 with 3 years follow-up  (n = 10,774) | Model 3 with 5 years follow-up  (n = 10,774) | Model 3 with competing risk of leaving NHS  (n = 10,766) | Model 3 with unnatural-cause deaths as a competing risk for natural-cause deaths  (n = 10,774) | Model 3 with cohort 50+  (n = 11,167) |
| --- | --- | --- | --- | --- | --- | --- | --- | --- |
| White British | Reference | Ref | Ref | Ref | Ref | Ref | Ref | Ref |
| Black African | 0.60 (0.50-0.71) | 0.64 (0.52-0.79) | 0.52 (0.33-0.81) | 0.68 (0.53-0.88) | 0.69 (0.56-0.86) | 0.64 (0.52-0.79) | 0.60 (0.48-0.75) | 0.63 (0.52-0.77) |
| Black Caribbean | 0.57 (0.52-0.61) | 0.55 (0.50-0.60) | 0.57 (0.47-0.69) | 0.56 (0.5-0.63) | 0.54 (0.49-0.60) | 0.55 (0.50-0.60) | 0.53 (0.48-0.58) | 0.55 (0.50-0.61) |
| South Asian | 0.70 (0.60-0.82) | 0.68 (0.57-0.81) | 0.64 (0.44-0.92) | 0.68 (0.54-0.86) | 0.64 (0.53-0.78) | 0.68 (0.58-0.81) | 0.67 (0.56-0.80) | 0.67 (0.56-0.80) |
| White Irish | 0.84 (0.75-0.93) | 0.85 (0.75-0.97) | 0.86 (0.67-1.11) | 0.90 (0.77-1.06) | 0.88 (0.77-1.01) | 0.86 (0.76-0.98) | 0.86 (0.76-0.98) | 0.86 (0.76-0.97) |
| Any other Asian background | 0.75 (0.62-0.90) | 0.73 (0.58-0.92) | 0.83 (0.55-1.26) | 0.78 (0.59-1.03) | 0.76 (0.60-0.97) | 0.73 (0.58-0.92) | 0.71 (0.56-0.90) | 0.73 (0.58-0.91) |
| Any other Black background | 0.63 (0.49-0.80) | 0.55 (0.41-0.74) | 0.40 (0.20-0.81) | 0.59 (0.41-0.86) | 0.60 (0.43-0.83) | 0.57 (0.42-0.77) | 0.56 (0.41-0.76) | 0.54 (0.40-0.73) |
| Any other ethnic group | 0.70 (0.61-0.82) | 0.62 (0.51-0.74) | 0.66 (0.47-0.92) | 0.70 (0.56-0.86) | 0.62 (0.51-0.76) | 0.62 (0.52-0.75) | 0.60 (0.50-0.73) | 0.62 (0.51-0.74) |
| Any other mixed background | 0.61 (0.29-1.30) | 0.42 (0.16-1.12) | (no deaths) | 0.31 (0.08-1.24) | 0.35 (0.11-1.09) | 0.42 (0.16-1.10) | 0.44 (0.17-1.15) | 0.37 (0.14-0.98) |
| Any other White background | 0.66 (0.59-0.73) | 0.63 (0.56-0.71) | 0.69 (0.54-0.89) | 0.67 (0.57-0.78) | 0.65 (0.57-0.75) | 0.64 (0.56-0.72) | 0.64 (0.56-0.72) | 0.63 (0.56-0.72) |
|  |  |  |  |  |  |  |  |  |
| *Covariates* |  |  |  |  |  |  |  |  |
| Age at diagnosis, squared† | 1.00 (1.00-1.00) | 1.00 (1.00-1.00) | 1.00 (1.00-1.00) | 1.00 (1.00-1.00) | 1.00 (1.00-1.00) | 1.00 (1.00-1.00) | 1.00 (1.00-1.00) | 1.00 (1.00-1.00) |
| Male gender | 1.51 (1.44-1.58) | 1.56 (1.47-1.64) | 1.57 (1.41-1.75) | 1.59 (1.48-1.7) | 1.58 (1.49-1.68) | 1.55 (1.47-1.64) | 1.55 (1.47-1.64) | 1.55 (1.47-1.64) |
| Index of multiple deprivation | 1.01 (1.00-1.01) | 1.01 (1.00-1.01) | 1.01 (1.00-1.01) | 1.01 (1.00-1.01) | 1.00 (1.00-1.01) | 1.01 (1.00-1.01) | 1.01 (1.00-1.01) | 1.00 (1.00-1.01) |
| Index MMSE Score† | 0.95 (0.95-0.96) | 0.95 (0.95-0.96) | 0.95 (0.94-0.96) | 0.96 (0.95-0.96) | 0.96 (0.95-0.96) | 0.95 (0.95-0.96) | 0.95 (0.95-0.96) | 0.95 (0.95-0.96) |

† Time interactions were 1.00 (1.00-1.00) because they were very small.
